# Extracellular Vesicles-ceRNAs as Ovarian Cancer Biomarkers: Looking into circRNA-miRNA-mRNA Code

**DOI:** 10.3390/cancers14143404

**Published:** 2022-07-13

**Authors:** Giuseppe Cammarata, Nadia Barraco, Ilaria Giusti, Valerio Gristina, Vincenza Dolo, Simona Taverna

**Affiliations:** 1 Institute of Translational Pharmacology (IFT), National Research Council of Italy (CNR), 90146 Palermo, Italy; 2 Section of Medical Oncology, Department of Surgical, Oncological and Oral Sciences, University of Palermo, 90127 Palermo, Italy; nadia.barraco@community.unipa.it (N.B.); valerio.gristina@unipa.it (V.G.); 3 Department of Life, Health and Environmental Sciences, University of L’Aquila, 67100 L’Aquila, Italy; ilaria.giusti@univaq.it (I.G.); vincenza.dolo@univaq.it (V.D.)

**Keywords:** extracellular vesicles, ovarian cancer, ceRNAs, microRNAs, circular RNAs, biomarkers

## Abstract

**Simple Summary:**

Patients with ovarian cancer have a very poor chance of long-term survival, usually due to advanced disease at the time of diagnosis. Emerging evidence suggests that extracellular vesicles contain noncoding RNAs such as microRNAs, piwiRNAs, circular RNAs, and long noncoding RNAs, with regulatory effects on ovarian cancer. In this review, we focus on ovarian cancer-associated circular RNA shuttled by extracellular vesicles as mediators of cancer progression and novel biomarkers in liquid biopsy. We propose a circular-RNA–microRNA-mRNA code that can reveal the regulatory network created by extracellular vesicles, noncoding RNAs, and mRNAs in ovarian cancer. Future research in this field will help to identify novel diagnostic biomarkers and druggable therapeutic targets, which will ultimately benefit patients.

**Abstract:**

Ovarian cancer (OC) is one of the most lethal gynecologic malignancies in females worldwide. OC is frequently diagnosed at an advanced stage due to a lack of specific symptoms and effective screening tests, resulting in a poor prognosis for patients. Age, genetic alterations, and family history are the major risk factors for OC pathogenesis. Understanding the molecular mechanisms underlying OC progression, identifying new biomarkers for early detection, and discovering potential targets for new drugs are urgent needs. Liquid biopsy (LB), used for cancer detection and management, consists of a minimally invasive approach and practical alternative source to investigate tumor alterations by testing extracellular vesicles (EVs), circulating tumor cells, tumor-educated platelets, and cell-free nucleic acids. EVs are nanosize vesicles shuttling proteins, lipids, and nucleic acids, such as DNA, RNA, and non-coding RNAs (ncRNAs), that can induce phenotypic reprogramming of target cells. EVs are natural intercellular shuttles for ncRNAs, such as microRNAs (miRNAs) and circular-RNAs (circRNAs), known to have regulatory effects in OC. Here we focus on the involvement of circRNAs and miRNAs in OC cancer progression. The circRNA-microRNA-mRNA axis has been investigated with Circbank and miRwalk analysis, unraveling the intricate and detailed regulatory network created by EVs, ncRNAs, and mRNAs in OC.

## 1. Introduction

Ovarian cancer is the fifth most common cause of cancer-related deaths among females and represents the second most lethal gynecological malignancy worldwide [1]. Ovarian cancer is a heterogeneous disease including both epithelial and mesenchymal cancers. Epithelial ovarian cancer (EOC, here referred to as OC) accounts for more than 90% of ovarian cancer; World Health Organization (WHO) classifies EOC into several morphological categories according to histopathology, immunohistochemistry, and molecular genetic analysis. EOC mainly includes endometrioid (EC), clear-cell (CCC), mucinous (MC), high-grade (HGSOC) and low-grade serous (LGSOC) carcinomas [2]. Among the EOC, HGSOC is the most common subtype [3]. Unfortunately, due to the lack of early symptoms and effective screening strategies, about 70% of such patients are diagnosed at an advanced stage, with metastasis to the bladder or uterus (stage II), abdomen (stage III), or beyond the peritoneal cavity (stage IV) [4]. Notwithstanding the improvement of surgery and novel antitumoral agents, the prognosis remains dismal, with very limited 5-year survival rates [3]. To date, although lacking specificity as a screening tool, CA-125 has been considered the only circulating biomarker used in the clinical setting for the longitudinal monitoring of patients with advanced OC undergoing standard systemic treatments [5]. Despite the different subtypes being distinct in etiology, morphology, genetic mutations, and prognosis, OCs are treated as a single entity. Heterogeneity is a key feature of these tumors, justifying, in part, the lack of successful treatments. With the development of molecular tools such as deep sequencing and epigenomics, new insights into the complexity of heterogeneity within these subtypes and individual patient tumors have been gained [6]. In the latest years, the increasing knowledge of molecular and genetic characteristics has undergone a paradigm shift favoring the introduction of targeted drugs in the therapeutic armamentarium of OC. It has been proved that about 50% of HGSOCs are deficient in the DNA homologous recombination repair pathway, with this defect being related to mutations (somatic or germline) of BRCA 1/2 genes or other genes involved in the homologous recombination deficiency (HRD) [7,8]. Genomic instability, a hallmark of cancer, is the result of altered DNA repair functions in tumor cells [9], inducing genomic rearrangements and nucleotide mutations. The failure to correct DNA damage in DNA repair defective cells, such as in BRCA1 and BRCA2 mutated backgrounds, is associated with an increased risk of OC [10]. Recent findings suggest the interdependence of genetic and epigenetic events in cancer pathogenesis. The alteration of epigenetic pathways may lead to acquiring of genetic mutations that, in turn, can alter the epigenome [11]. The genomic instability can be blocked by the inhibition of Poly (ADP-ribose) Polymerase (PARP) enzymes, which are used as clinically approved biological targets playing an integral role in single-strand DNA break repair via the base excision pathway [12]. Based on the mechanism of “synthetic lethality”, which selectively kills tumor cells with deficient homologous recombination, PARP inhibitors (PARPi) have been approved as the first targeted drugs in OC therapeutic management [13]. Nevertheless, a range of resistance mechanisms to such treatments, such as reversion of secondary mutations restoring the wild-type BRCA reading frame or protein expression, have already been documented in the literature [14]. In this fascinating scenario, the growing LB family (non-coding RNAs, extracellular vesicles, circulating tumor cells, cell-free DNA, and RNA) might be crucial for diagnostic and prognostic purposes while retaining important implications for therapeutic response to PARPi [15,16]. In this field, several studies exploring next-generation sequencing and bioinformatics analyses have affirmed the compelling role of circulating nucleic acids in the pathogenesis and progression of many human diseases, including OC [17]. In this complex landscape, the discovery of novel and reliable biomarkers with prognostic and predictive significance is urgently warranted, representing an unmet clinical need for enhancing the diagnosis and prognosis of OC patients.

In this review, we focus on promising OC-associated ncRNAs shuttled by EVs as mediators of cancer progression and emerging biomarkers in OC liquid biopsy. The circRNA–microRNA code identified by Circbank, and miRwalk analysis, reveals the intricate and detailed regulatory network created by Evs, ncRNAs, and messenger RNAs (mRNAs) in OC.

## 2. Search Strategy

The search was conducted in PubMed, limited to articles published between 1 January 2005 and 31 May 2022. Research papers and review articles were included in the evaluation. Only articles published in the English language were considered. Relevant articles were searched as follows: (“Ovarian cancer” AND “*”), where “*” stands for: (i) “extracellular vesicles” OR “exosomes” OR “microvesicles”; (ii) “non coding RNA; (iii) “microRNA” OR “miRNA” or “miR”; (iv) “circular RNA” OR “circRNA”; (v) “BRCA 1/2” OR “BRCA”; (vi) “Poly (ADP-ribose) Polymerase (PARP)” OR “PARP”; (vii) “genomic instability”; (viii) “epigenome OR epigenetic”. Articles were sorted by relevance and screened for suitability to the aim of the review. Currently, the data reported in the literature do not allow to specifically address the different ncRNA alterations to the subtypes of ovarian cancer; thus, this review is focused on epithelial ovarian cancers (OCs).

## 3. Extracellular Vesicles

Extracellular Vesicles (EVs) are nanoscale membrane vesicles released by all cytotypes in physiological and pathological conditions. Although EVs have been described, for the first time, as a device to eliminate “cellular garbage” in mammalian reticulocyte maturation [18], in the last two decades, the interest of the scientific community in the EV field has grown exponentially [19]. EVs are classified into two main families, exosomes and microvesicles, which differ in biogenesis, size, and mechanism of release [20]. Microvesicles (diameter of 200–1000 nm) are released directly by the outward budding of the plasma membranes by all cytotypes. Exosomes (diameter of 30–200 nm) originate from the endo-lysosomal compartment of multivesicular bodies after the fusion of intraluminal vesicles with the plasma membrane [21,22,23]. Both these vesicles are widely present in various biofluids, such as blood, urine, cerebrospinal fluid, saliva, amniotic fluid, breast milk, gastric juice, and malignant effusions, such as pleural and ascitic fluids [24,25]. Moreover, it was demonstrated that cancer cells release a higher amount of EVs compared with normal cells [26]. EVs are internalized by target cells as intact vesicles surfing on filopodia [27] or through different mechanisms, including direct fusion with plasma membranes, receptor-ligand binding, micropinocytosis, phagocytosis, actin polymerization [28,29]. EVs transport a plethora of bioactive molecules, including proteins, lipids, and nucleic acids, such as DNA and RNAs that mirror the status of cells and tissues of origin [30,31,32]. The rapid development of high-throughput technologies led to the identification of complete EV molecular composition. Several databases have been created to integrate and organize this information, including Vesiclepedia, ExoCarta, and EVpedia [33,34,35]. Among these databases, currently, Vesiclepedia contains 349,988 protein entries, 27,646 messenger RNAs, 10,520 microRNAs, and 639 lipids, which are derived from 1254 studies in 41 different species [36]. Several non-coding RNAs, with regulatory effects, including microRNAs (miRNAs), P-Element induced wimpy testis interacting (PIWI) RNAs (piRNAs), circular-RNAs (circRNAs), and long noncoding-RNAs (lnc-RNAs), have been described in EVs [37,38,39,40,41,42]. The high number of EV cargos reflects the number of targets that can be modulated by EVs and highlights their importance as biomarkers in LB [43,44]. EVs encapsulate their cargo into the phospholipid bilayer and transport it with high stability, conferring resistance to degradation and a long half-life. EV proteins maintain their biological functions, including antigen presentation, protein cleavage, and pathway activation. Similarly, the nucleic acids, shuttled by EVs, maintain their bioactivity: mRNAs can be translated into target cells, and regulatory ncRNAs, as miRNAs and circRNAs, can mediate RNA-silencing, allowing a dynamic and fine regulation of gene expression [45]. Furthermore, EVs play a key role in intercellular communication and have been associated with several physiological and pathological functions [46,47]. EVs released by cancer cells are involved in angiogenesis [21,29,48,49], immune system escape, microenvironment remodeling, premetastatic niche formation (PMNF) and metastasis dissemination, and resistance to therapy, all features that support tumor progression [50,51,52]. Recently, it was reported that EVs could be bioengineered utilizing genetic methodology to design and produce EVs with innovative functionalities and properties based on knowledge of their biogenesis, release, and uptake pathways [53]. Several reports also described EV-versatility in translational medicine, such as applications of EVs in diagnosis, prevention, and treatment of disease. One of the major potential clinical applications of EVs is the use of their cargos as biomarkers [25].

### 3.1. Roles of Extracellular Vesicles in Ovarian Cancer

**Figure 1:**
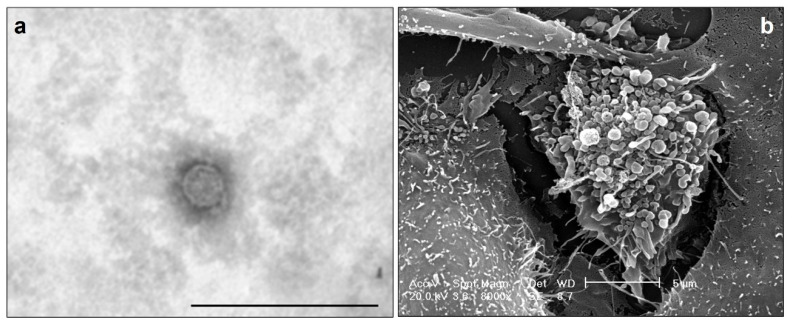
Electron Microscopy analysis. Ultrastructural morphology of human ovarian cancer isolated EV by Transmission Electron Microscopy (TEM). The size bar is 250 nm (**a**). Morphology of human ovarian cancer cells, releasing EVs imaged by Scanning Electron Microscopy (SEM), Magnification 8000x. The size bar is 5 µm (**b**). Personal data.

The peculiarity of OC is the peritoneal cavity invasion through the ascites, which contains mesothelial and immune cells, detached tumor cells, and OC-associated EVs (OC-EVs). The peritoneum is the homing site for OC cells, and the exfoliation of cancer cells from their primary location is accompanied by their morphological reorganization [54]. Like other cancer cells, human OC cells release a great number of EVs, typically enclosed in a phospholipid bilayer (Figure 1a), that can be quite heterogeneous in size (Figure 1b), comprising both small and large EVs.

**Figure 2:**
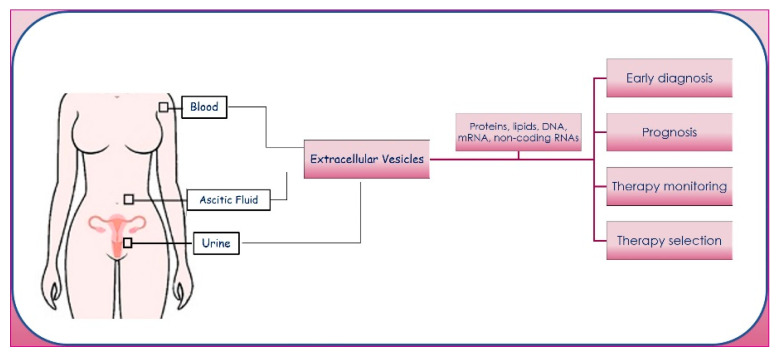
Schematic representation of ovarian cancer EVs origin, composition, and biological functions.

Generally, the exchange of molecular signals is an important feature in cell invasion and metastasis, and EVs play a crucial role in cell–cell communication; the pharmacological inhibition of EV uptake at metastatic sites or the decrease in EV release by cancer cells inhibits the PMNF [55]. EVs can be isolated from plasma [56,57], ascites [58,59], and urine [60,61] of patients with OC. EVs in these complex biofluids have a pivotal role in cancer progression, and their cargos may act as useful biomarkers for diagnosis, prognosis, therapy selection in different disease statuses, and monitoring response to therapies (Figure 2). Recent findings indicate that EVs have a superior standing compared to nucleic acids and proteins freely circulating in the blood as a tool for LB [62].

#### 3.1.1. Cancer Progression

**Figure 3:**
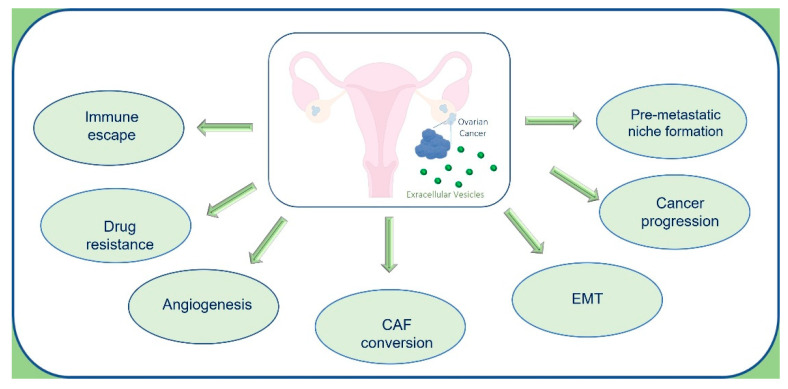
Schematic representation of the pleiotropic roles of ovarian cancer EVs in tumor progression.

OC-EVs have a pleiotropic role in cancer progression (Figure 3), acting as crucial mediators of intercellular communication by transferring bioactive molecules, which can reprogram the phenotype of target cells [22,63]. OC-EVs, in addition to cargos typically present in tumor-derived EVs (tetraspanins, heat shock, membrane proteins, antigens, and enzymes), contain unique protein signatures specific to OC. Among these proteins, OC-EVs transport Nanog, a transcription regulator involved in cancer cell proliferation and self-renewal of cancer stem cells [64]. OC-EVs also transfer CD44 to mesothelial cells inducing epithelial-mesenchymal transition (EMT), downregulating E-cadherin, and inducing MMPs expression, that in turn promotes OC invasion and metastasis through degradation of extracellular matrix (ECM) [65]. EVs collected by ascitic fluid (AF-EVs) from OC patients transport CD24 and EpCAM, which induce cell migration, increasing tumor invasiveness [66]. Moreover, AF-EVs are enriched in ZBED2, ZBTB20, ABCC3, UHMK1, proteins with a role as drug transporter or cell cycle regulators, and a low amount of transgelin and MARCKS with respect to benign EVs [58]. AF-EVs also contain soluble L1 (adhesion molecule), matrix metalloproteinase MMP-2, membrane-type matrix metalloproteinase (MT1-MMP), and urokinase-type plasminogen activator (uPA), which promote cancer cell migration [67].

AF-EVs contribute to angiogenesis via soluble E-cadherin (sE-cad); this protein contained in EVs heterodimerizes with VE-cadherin on endothelial cells and induces a sequential activation of β-catenin and NFκB signaling, stimulating endothelial cell migration, permeability, and neovascularization [68]. OC-EVs educate the cells of the pre-metastatic niche to promote metastasis, creating a favorable microenvironment for metastatic cells [64]. In peritoneal metastases, cancer cells are exposed to hypoxic conditions since they detach from the primary tumor and float into the peritoneal cavity without vascular spraying. Hypoxia induces cancer cells to acquire a more aggressive phenotype that contributes to metastasis formation [69]. In hypoxic conditions, in several cancer types, including OC, cells release a high amount of EVs with respect to normoxic cells in a hypoxia-inducible factor (HIF)-1α-dependent and independent manner. These EVs deliver oncogenic signals and play key roles in proliferation, invasion, stemness, angiogenesis, drug resistance, and immune evasion [18]. In response to low oxygen concentrations, OC promotes vessel recruitment and, activating transcription factor 2 (ATF2), metastasis-associated protein 1 (MTA1), and CD147 contained in OC-EVs, induces angiogenesis and vascular permeability [70,71]. OC-EVs can also convert normal fibroblasts into activated cancer-associated fibroblasts (CAFs); CAFs, in turn, create a microenvironment that induces angiogenesis, malignant cell proliferation and invasion, tumor growth, immunosuppression, and drug resistance [72,73,74,75].

EVs released by ovarian CAFs (EV-CAFs) are enriched in TGFβ1 compared to normal fibroblasts. EV-CAFs promote EMT in cancer cells through the activation SMAD pathway, inducing OC cell migration and invasion [76].

It was demonstrated that OC-EVs contain epidermal growth factor receptor (EGFR) [77,78]. EGFR signaling is one of the most studied cellular pathways involved in tumor progression. EGFR is also useful as a target of drugs for the development of cancer therapies. EVs shuttle EGFR and EGFR ligands, between tumor cells and local or distant target cells, as molecules functionally active [79,80].

Several EGFR ligands, such as EGF, transforming growth factor-α (TGFα), amphiregulin (AREG), and epiregulin (EREG), have been described in EVs. They regulate EGFR function, activating signal transduction that leads to OC progression [81,82,83]. A pilot study using an immunocapture chip has identified a significant difference in EGFR expression on plasma derived EVs between patients with OC and controls. This system allowed to test EGFR, HER2, CA125, FRa, CD24, EpCAM, CD9, and CD63 in OC-EVs with a diagnostic power in early cancer diagnosis [84]. EVs, with their small size, act as a single-point, directional delivery mechanism for the activation of EGFR family receptors [82]. The transmembrane EGFR ligands displayed on EVs active a signaling pathway recently indicated as ExTRAcrine (Exosomal Targeted Receptor Activation) mechanism [79].

#### 3.1.2. Immunomodulatory Effects

EVs function as carriers of different immunomodulatory molecules for pro-tumorigenic activities. OC-EVs facilitate the immune escape, supporting OC progression. OC-EVs derived from primary tumors and ascites, indeed, shuttle immune-modulatory biomolecules that can induce immune escape. AF-EVs induce the release of interleukin-6 (IL-6) from monocytic precursor cells via toll-like receptor (TLR) pathway activation [85]. Then, IL-6 activates the signal transducer and activator of the transcription 3 (STAT3) pathway in immune cells, stromal cells, and tumor cells, which supports the immune escape of cancer cells [86]. EVs from ascites and plasma of OC patients can suppress T cells carrying arginase-1 (ARG-1); these EVs travel through the draining lymph nodes and are internalized by dendritic cells to inhibit antigen-specific T cell proliferation [87]. OC-EVs contain FasL that can also induce T cell suppression via Janus kinase (JAK) signaling [88]. OC-EVs, internalized by natural killer (NK) cells, damage the NK2D-mediated cytotoxicity inducing immunosuppression [89]. AF-EVs induce a rapid and reversible T cell arrest via GD3, a ganglioside expressed on the surface of AF-EVs, acting on their T-cell receptor (TCR) [90,91]. EVs are also involved in macrophage polarization. Macrophages are multifunctional antigen-presenting cells divided into two polarized classes: pro-inflammatory (M1) and anti-inflammatory (M2). Tumor associate-macrophages (TAMs) are M2 subtypes and permeate malignant tissues; they are associated with poor prognosis, promoting tumor growth and metastasis [92]. TAMs secrete IL4, IL-5, and IL-6, which promote angiogenesis, matrix remodeling, and immune system suppression [93]; they also contribute to PMNF by secreting TGF-β, SDF-1, and VEGF via the STAT3 signaling cascade. When OC-EVs are phagocytosed by undifferentiated macrophages, they undergo M2 polarization via the suppressor of cytokine signaling (SOCS)4/5/STAT3 pathway [94]. OC-EVs mediate the interaction between TAMs and T cells, generating an immune-suppressive microenvironment that facilitates OC progression and metastasis. TAM-derived exosomes shuttle miRNAs, such as miR-29a-3p and miR-21-5p, to synergistically induce the Treg/Th17 cell imbalance through direct targeting of STAT3 in CD4+ T cells [95].

#### 3.1.3. Drug Resistance

EVs are involved in chemoresistance; although most patients initially respond to chemotherapy, about 70–80% of tumors recur and become resistant to treatment [96,97]. EVs contain a mix of biological effectors that can contribute to drug resistance in OC. The amount of EVs secreted by chemo-resistant OC cells is estimated at 2.6 times that of drug-sensitive cells, and the more aggressive OC cell line release a higher amount of EVs [98,99]. Moreover, EVs released by drug-resistant cells of different tumors can transmit the resistance to sensitive cells. It was demonstrated that OC-EVs transport plasma gelsolin (pGSN); in chemo-resistant conditions, increased secretion of EV-pGSN by OC cells induced apoptosis in CD8+ T cells and reduced IFNγ secretion, resulting in high GSH production and resistance to cis-diaminedichloroplatinum (CDDP)-induced death in OC cells [100]. Moreover, STAT3 and FAS oncoproteins transported by AF-EVs significantly increase the resistance to cisplatin [101]. These data indicate the important role of OC-EVs in drug resistance of OC, suggesting the pleiotropic effects of EVs in OC progression and their power as emerging biomarkers in OC liquid biopsy.

## 4. Noncoding RNAs in Ovarian Cancer

Approximately 75% of the human genome is transcribed into RNA, while only 3% is transcribed into protein-coding mRNAs [102]. According to the length, shape, and location, ncRNAs have been divided into different classes. Among them, miRNAs, piRNAs circRNAs, and lncRNAs are the four major ncRNA classes with distinct functions in human diseases. Abundant evidence has shown that ncRNAs are frequently deregulated and play crucial roles in cancer; they can work as oncogenes or suppressors to regulate cancer initiation and progression. Many ncRNAs can be abnormally released from cancer cells via EVs circulating in the blood or other biological fluids. Since EV-ncRNAs mirror the status of parental cells, they can act as diagnostic markers or prognostic indicators. EVs shuttled ncRNAs with regulatory effects on target cells. In order to achieve these functions, the cells are able to induce a selective packaging of EV-ncRNAs, through a purposeful rather than passive process. The ncRNA sorting is driven by different mechanisms, including the interactions between RNA-binding protein (RBP) and specific ncRNA-binding motifs capable of exerting selectivity over the ncRNAs shuttled into EVs [103]. One of the primary causes of the deregulation of ncRNAs can be attributed to epigenetic changes. In turn, ncRNAs can modify the epigenome by interacting with epigenetic regulators or modifying their expression, thereby establishing a complex network that is perturbed during carcinogenesis. This complex network could be altered in response to environmental changes and/or stress responses, including exposure to toxic pollutants or carcinogenic agents [104,105]. Emerging studies are starting to link distinct types of mutations in ncRNA genes with cancers. However, the precise mechanism by which mutations in ncRNAs contribute to the pathogenesis of the disease remains unclear. Many genetic and somatic mutations impacting miRNA-mRNA interactions have been associated with various cancers [106]. In addition, miRNAs can also interact with other ncRNAs, such as circRNAs and lncRNAs [107,108]. The lncRNAs, circRNAs, and mRNAs with common miRNA target sites compete for miRNA binding and form a complex network of interaction and regulation, known as the competing endogenous RNA (ceRNA) network [109]. Mutations in mature miRNA sequence on its target sites and ceRNAs may alter miRNA-ceRNA interactions and rewire the ceRNA network [110]. In human cancer, shortening of the 3′-untranslated region (3′-UTR) through alternative polyadenylation (APA) is widespread, affecting thousands of genes [111]. Since 3′-UTR contains miRNA target sites, mRNAs with shortened or lengthened 3′-UTRs may effectively diversify transcriptomic dynamics in diverse pathological conditions such as cancer [112]. An emerging class of ncRNAs involved in cancer progression is represented by piRNAs. The piRNAs are ncRNAs of approximately 24–31 nucleotides; they have a 5′ terminal uridine or tenth position adenosine bias, lack clear secondary structure motifs and interact with PIWI, which are nuclear RNA-binding proteins [113]. piRNAs were initially described in germline cells, emerging evidence shows they are expressed in a tissue-specific manner in multiple human somatic tissues and cancer. The classical function of PIWI/piRNAs is to maintain genomic integrity by repressing the mobilization of transposable elements and regulating the expression of downstream target genes via transcriptional or post-transcriptional mechanisms. Increasing functional evidence supports the involvement of piRNAs in the regulation of epigenetic changes in tumorigenesis, increasing functional evidence supports the involvement of piRNAs in the regulation of epigenetic changes in tumorigenesis [114]. Abnormal expression of piRNAs is emerging as a crucial regulator in cancer cell proliferation, apoptosis, invasion, and migration. In OC the study of piRNA expression and its pathophysiological significance remains exploratory. It was demonstrated that piR-52207 and piR-33733 were increased in OC. piR-33733 targets LIAS3′ -UTRs, whereas piR-52207 binds ACTR10 and PLEKHA5′-UTRs and 5′-UTRs, leading to increased anti-apoptotic and decreased pro-apoptotic proteins. Thus, piR-52207 and piR-33733 promote OC oncogenes via involvement in multiple cell-signalling pathways at the post-transcriptional level, supporting them as potential therapeutic targets for OC [115]. Moreover, lncRNAs are considered emerging players in cancer metastasis and potential diagnostic biomarkers for personalized oncology. LncRNAs have a length of 200–100,000 nucleotides, lack a complete open reading frame (ORF), are transcribed by RNA polymerase II, capped, and polyadenylated at the 5′ and 3′ ends, respectively [116], and rarely encode short functional peptides. ENCODE project reports that the human genome encodes more than 28,000 distinct lncRNAs [117]. This class of lncRNAs is involved in several mechanisms such as modification of histones, chromatin remodeling, and regulation of transcriptional and post-transcriptional processes. They can act as enhancers, scaffolds, sponges binding various miRNAs, or even precursors of some miRNAs [118]. LncRNAs can function both as oncogenes or tumor-suppressors; aberrant expression of oncogenic lncRNAs alters cell signaling cascades, affecting cell proliferation, survival, invasion, angiogenesis, metastasis, and promoting tumor progression [119]. LncRNAs may mediate resistance to cancer-specific chemotherapy, and they can also be used as a potential diagnostic tool. Recent studies have shown that lncRNAs can be packaged into EVs to mediate extracellular communication with local or distant cells. EV-lncRNAs are internalized by recipient cells via direct fusion, endocytosis, and receptor-ligand binding. EV- lncRNAs in target cells can act as (I) ceRNAs that interact with miRNAs and interfere with their function, (II) scaffold that recruits and binds proteins regulating their activity, (III) decoy that interacts with transcription factors (TFs) altering transcriptional regulation, and (IV) guide to promote gene expression by recruiting TFs to a gene promoter [41]. The involvement of several lncRNAs in initiation, progression, and drug resistance of OC has been described. The lncRNAs are involved in regulation of the expression of protein-coding genes through direct binding along lncRNA/miRNA/mRNA axis, with the direct participation of miRNA [120]. An increasing number of studies shows that EV-lncRNAs can sponge miRNAs to regulate target gene expression and can bind proteins to affect their phosphorylation or ubiquitination, regulating their expression and activity [41]. The well-known lncRNA Metastasis-associated lung adenocarcinoma transcript 1 (MALAT1) has also been identified in OC-EVs and it is associated with cancer angiogenesis and metastasis. OC-EVs shuttle MALAT1 to endothelial cells inducing proangiogenic activity. Clinically, high levels of EV-MALAT1 in OC patients’ serum are highly correlated with an advanced and metastatic phenotype of OC. EV-MALAT1 is an independent predictive factor for OC overall survival [121]. Another lncRNA, SOX2-OT, contributes to OC progression by miR-181b-5p/SCD1 signalling. SOX2-OT expression levels are upregulated in EVs collected by OC patients’ plasma. SOX2-OT sponges miR-181b-5p, that in turn can target SCD1 in OC cells. Overall, SOX2-OT, miR-181b-5p, and SCD1 can represent potential targets for OC treatment [122]. Recently, it was demonstrated that LncRNA SPOCD1-AS contained in OC-EVs can remodel mesothelial cells to promote peritoneal metastasis via interacting with G3BP1. LncRNA SPOCD1-AS is secreted by OC cells and shuttled to the recipient mesothelial cells, via EVs, inducing mesothelial-mesenchymal transition (MMT) binding to G3BP1 in mesothelial cells. To confirm the role of LncRNA SPOCD1-AS, a cell-penetrating interfering peptide that blocks SPOCD1-AS/G3BP1 interaction has been created. This peptide inhibits MMT of mesothelial cells in vitro and suppressed OC peritoneal metastasis in vivo [123]. This review is focused especially on circRNA/miRNA/mRNA axis and the epigenetic modifications that can induce genomic instability and OC progression.

## 5. Circular-RNAs in Ovarian Cancer

Noncoding RNAs (ncRNAs) can be classified as housekeeping and regulatory ones. Housekeeping ncRNAs are involved in the general maintenance of normal cell functionalities and are ubiquitously expressed, whilst regulatory ncRNAs have been identified as key modulators of gene expression in different cellular processes [124]. Among these ncRNAs, miRNAs and circRNAs free in plasma or contained in EVs have been described in OC [125]. CircRNAs were first considered as functionless by-products derived from aberrant RNA splicing caused by errors during post-transcriptional processing; nowadays, circRNAs are considered a new class of ncRNA with high regulatory potential [126]. CircRNAs were first discovered in 1990 when observing that exons of a tumor suppressor gene after their splicing were joined in a different order than their genomic sequence. They are generated for the back-splicing process from linear pre-messenger RNAs when 3′ and 5′ ends are ligated to form a continuous loop and covalently closed [127]. CircRNAs are highly resistant to RNAse activity since they lack 5′ and 3′ ends; thus, they are more stable and have a longer half-life than canonical linear isoforms [128]. Recently, high-throughput technologies have revealed the abundance and diversity of circRNAs. The principal functions of circRNAs are miRNA inhibition, interaction with RNA-binding proteins, and regulation of gene expression [129]. CircRNAs are more than 100.000 per cell and mainly act as miRNA sponges by protecting target genes from repression by miRNAs; one circRNA can sponge different miRNAs establishing an intricate and precise regulatory network [130]. They have multiple regions/binding sites termed miRNA response elements (MREs), that function as competitive endogenous RNA (ceRNA) to regulate the biological activity of miRNAs and restore their inhibitory function in the target gene [131]. CircRNAs are aberrantly expressed in cancer tissues and have several advantages over canonical linear RNAs as cancer biomarkers [132]. An increasing number of studies reported the abnormal expression of circRNAs in OC, suggesting the relevance of these ncRNAs, as essential regulatory factors, in OC tumorigenesis and progression. In 2015, circRNAs were described in EVs for the first time and high-throughput technologies, such as genome-wide RNA-seq analyses, showed that they were enriched in EVs by 2-fold in comparison with parental cells [133]. Recent findings indicate that circRNAs have a dual role in OC; the dysregulation of specific circRNAs contributes to promoting or limiting OC tumorigenesis, progression, and metastasis. Many circRNAs have been described in this regard: it was demonstrated that high expression of circWHSC1 in OC promotes tumorigenesis by sponging miR-145 and miR-1182 and that circWHSC1 shuttled by EVs induces tumor metastasis; circMUC16 promotes autophagy of OC via interaction with ATG13 and miR-199a, and its expression has been linked to the progression in stage and grade of OC [134]; circRNA forkhead box O3 (circ-Foxo3) promotes OC progression through EV-mediated intercellular interaction to target miR-422a/PLP2 axis and can also be considered a potential biomarker for OC [135]. CircRNA051239 expression is increased in tissues and EVs of OC patients’ plasma; it acts as ceRNA by sponging miR-509-5p to facilitate Serine protease 3 (PRSS3) expression. PRSS3 is a member of the trypsin family of serine proteases involved in tumor growth and metastatic progression of OC [136]. Circ-0001068 is significantly increased in EVs of OC patients compared with healthy controls; it is also reported to be shuttled by EVs into T cells and to induce PD1 expression by sponging miR-28-5p to promote tumor immune invasion [137]. Furthermore, Cdr1as, known as circRNA sponge for miR-7 (ciRS-7), formed by reverse splicing of the antisense strand of the cerebellar degeneration-associated antigen 1 (CDR1) gene, is involved in OC drug resistance. Cdr1as was observed to be downregulated in cisplatin-resistant patient tissues and cell lines; its overexpression inhibits cell proliferation and promotes the cisplatin-induced cell apoptosis in OC cells. Cdr1as inhibition promotes miR-1270 expression, which displays its role via modulating the Suppressor of Cancer Cell Invasion (SCAI) expression. It was also demonstrated that Cdr1as is downregulated in serum exosomes from cisplatin-resistant patients; these data indicate that Cdr1as sensitizes OC to cisplatin by regulating the miR-1270/SCAI signaling pathway [138]. CircHIPK3 has an enhanced expression level in OC patients; it can modulate VEGF expression through miR-7 inhibition affecting OC cell proliferation and apoptosis [139]. Several other circRNAs have a regulatory role in OC progression: circ_0061140 functions as a ceRNA of miR-370, promoting OC cell proliferation and metastasis, through regulation of miR-370/FOXM1 pathway mediating EMT [140]; circ_0051240 promotes cell proliferation, migration and invasion in OC inhibiting miR-637/KLK4 axis [141]; circ-CSPP1, sponging miR-1236-3p, reduces the inhibitory effect of miR-1236-3p on ZEB1, which in turn promotes EMT and OC development [142]; circEPSTI1 is overexpressed in OC and regulates EPSTI1 expression sponging miR-942, promoting OC progression [143]; circPIP5K1A contributed to OC progression via targeting the miR-661/IGFBP5 axis [144]; circGFRA1 regulated GFRA1 expression and OC progression by sponging miR-449a [145]. Other circRNAs can inhibit OC progression: it was reported that CircPLEKHM3 acts as a tumor suppressor sponging miR-9 to regulate the expression of BRCA1, DNAJB6, and KLF4, and consequently inactivate AKT1 signaling [146]; circ_0078607 can suppress OC progression by sponging oncogenic miR-518a-5p to induce FAS expression, inhibiting proliferation and invasion and promoting apoptosis of OC cells [147]; circEXOC6B suppressed the growth of OC cells by upregulating RSU1 via sponging miR-421 [148]; circ_0078607 could serve as a sponge of miR-32-5p to regulate SIK1 expression, thus inhibiting OC progression [149]. Another circRNAs, circ-ITCH, can inhibit OC progression by suppressing cell proliferation and apoptosis, sponging miR-10a [150]. Recently, it was reported that circRNAs are also involved in OC drug resistance [151]. Circ_0025033 downregulation impaired paclitaxel (PTX) resistance and malignant activities of PTX-resistant OC cells by regulating miR-532-3p/FOXM1 network: it upregulates FOXM1 expression by targeting miR-532-3p. Circ_0025033 and FOXM1 are highly expressed, while miR-532-3p is low expressed, in OC cells. Circ_0025033 knockdown reduces PTX resistance, suppresses migration and invasion, and promotes apoptosis of PTX-resistant cells [152]. Moreover, it was demonstrated that circ_C20orf11 expression in OC is abnormally upregulated and enhances cisplatin (DDP)-based chemotherapy resistance. Circ_C20orf11 promotes EV-mediated M2 macrophage polarization by inhibiting miR-527/tyrosine 3-monooxygenase/tryptophan 5-monooxygenase activation protein zeta (YWHAZ) signaling, in turn promoting DDP resistance in vitro and in vivo [153]. Overall, these findings show that circRNAs have a pivotal role in the regulation of the main steps of cancer progression. Moreover, they are potent cancer biomarkers and can be considered the new frontier of LB.

## 6. MicroRNAs in Ovarian Cancer

miRNAs are small (19–22 nucleotides) ncRNA molecules that bind a complementary sequence of 3′ UTR of a target mRNA, inducing target degradation or translation inhibition, thus regulating gene expression and controlling many biological processes, such as proliferation, invasion, cell cycle progression, apoptosis, and immune response [154,155]. The extensive dysregulation of miRNA expression leads to cancer, and the mechanisms responsible for this condition involve genetic and epigenetic alterations in transcriptional and post-transcriptional machinery, DNA point mutation, chromosomal alterations, and translational modulation [156]. Recent studies have shown that 30–60% of human protein-coding genes are regulated by miRNAs [157]. miRNAs also interact with other families of regulative RNAs, such as circRNAs [158]. CircRNA-miRNA-mRNA axis is closely related to cancer progression. According to ceRNA theory, circRNAs may act as mRNA expression regulators by sponging miRNAs. Since 2008, miRNAs have been identified in plasma and serum as cell-free circulating miRNAs or encapsulated in EVs [159]. Several studies have reported that EVs of different origins contain a unique expression profile of miRNAs, mirroring the state of parental cells. Aberrant expression of miRNAs is implicated in the progression of cancer, including OC; their levels can be up or downregulated in OC cells, and plasma samples of OC patients, and their abnormal expression is highly associated with OC metastasis [160]. Deregulated miRNAs can modulate the main OC-related pathways such as PI3K/Akt, Wnt/β-catenin [161], mTOR [162], MAPK [163], and EGFR [164]. Among the OC downregulated miRNAs, miR-145 suppresses TRIM2 expression; when Bim is phosphorylated by ERK, TRIM2 enhances Bim degradation in proteasomes, inhibiting apoptosis [165]. MiR-506 is considered a tumor suppressor miRNA that directly targets CDK4 and CDK6. FOXM1 is phosphorylated and activated by these CDKs, so miR-506 downregulation leads to overexpression of CDK4/6 and activation of FOXM1, enhancing OC progression [166,167]. Moreover, the suppression of miR-222-3p induces PDCD10 expression that, in turn, increases cancer metastasis by downregulating E-cad and enhancing Vimentin, promoting EMT and β-catenin/Wnt-mediated cell migration [168]. Bi et al. reported that an increase in miR-126-5p, mediated by Methyltransferase-like 3 (METTL3), promotes OC progression via PTEN-mediated PI3K/Akt/mTOR pathway [162]. Among the miRNAs upregulated in OC, miR-205 is associated with metastatic progression in OC patients and is enriched in cancer-adjacent endothelial cells; its upregulation is positively correlated with high microvessel density in OC patients. miR-205 is also increased in the serum of OC patients, and high levels of miR-205 in EVs are associated with OC metastasis [169]. Moreover, it was demonstrated that miRNAs contained in OC-EVs could enhance the growth and migration of OC cells. Furthermore, it was reported that AF-EVs contain high levels of miR-200c-3p, miR18a-5p, miR1246, and miR1290 and a low amount of miR-100-5p as compared to EVs isolated from a benign ascitic fluid; these vesicles enhance the migration of OC spheroids towards omental fat [58]. Overall, these reports indicate the key role that miRNAs, regulated upstream by circRNAs, play in the modulation of genes involved in OC progression.

## 7. circRNA–microRNA-mRNA Code in Ovarian Cancer

**Figure 4:**
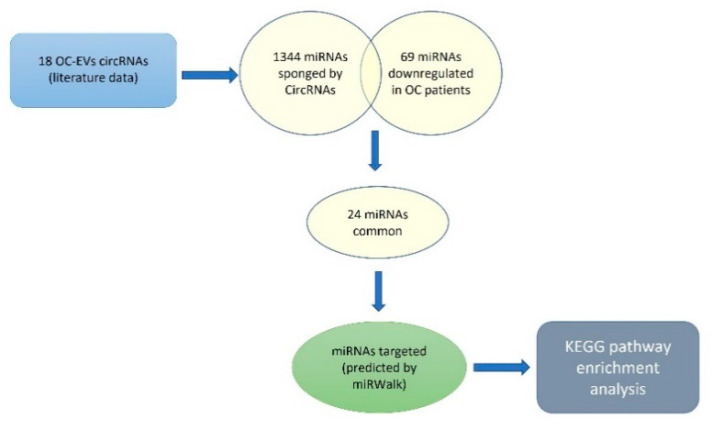
In silico analysis workflow.

There is evidence to suggest that circRNAs can be linked with cancer, owing to their interactions with miRNAs that regulate cancer-related gene expression [170]. To gain deep insight into the autocrine effect of circRNAs shuttled by EVs on OC cells, we elaborated on a potential interaction of EV-circRNAs on miRNAs in OC cells and mRNA targets (Figure 4).

**Figure 5:**
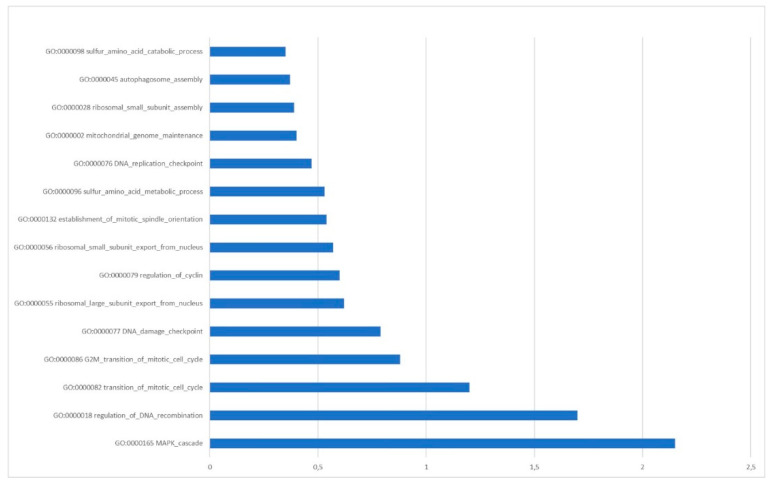
The top 15 Enriched GO Pathways from Predicted Target Genes of 24 selected miRNAs searched (MiRWalk, version January 2022).

Starting from the literature data we selected, 18 circRNAs were frequently found upregulated in OC (Table 1). Then, using the circBANK online tool, we identified 1344 miRNAs predicted to be sponged by the selected circRNAs. Matching the 1344 miRNAs with 69 miRNAs known, from clinical studies, to be downregulated in OC patients, 24 common miRNAs result (Table 2). The functional analysis revealed that the mRNAs targeted by these miRNAs are involved in pathways highly related to the genomic stability maintenance process (Figure 5).

Our in-silico analysis also indicates that among the biological processes involved in genomic stability, the maintenance of mitochondrial genome, DNA damage response, and DNA repair via homologous recombination are modulated by the analyzed cirRNA-miRNA code. NcRNAs regulate gene expression through epigenetic mechanisms and act as signaling molecules to regulate essential cellular and biological processes [194]. Maintenance of the mitochondrial genome is essential for the correct cellular function, and ncRNAs are involved in the epigenetic regulation of mitochondrial gene expression. The regulation of mitochondrial DNA by epigenetic changes facilitates crosstalk between the nucleus and mitochondria, leading to the maintenance of cellular health and homeostasis; the failure of this process can lead to various cancers, including OC [195]. In addition, the preservation of genetic code is crucial for healthy cells; several molecular pathways are activated by the cell to recognize and repair DNA damage [196]. The failure of a DNA damage response can lead to the introduction of mutations that drive normal cells to abnormal proliferation and other dysfunctions, often leading to cancer. BRCA2 is a guardian of genome integrity by promoting homologous recombination-based repair of DNA breaks. Since BRCA2 controls the replication fork during the DNA damage response, its germline mutations predispose to breast and ovarian cancer [197]. Defective DNA repair via homologous recombination (HR) is also a critical vulnerability of OC; about 50% of OC show defective DNA repair via HR due to genetic and epigenetic alterations of HR pathway genes [198]. Increasing genomic instability with radiotherapy and chemotherapy has been an efficient but nonselective way of killing cancer cells. Precision medicine has revolutionized cancer therapy by introducing the concept of cancer-cell-selective targeting [199]. PARPIs represent the first example of precision medicine as drugs targeting DNA damage response used in clinic management. PARPIs act through synthetic lethality with mutations in DNA repair genes and were approved for the treatment of BRCA mutated ovarian and breast cancer [200]. The discovery of new agents targeting the circRNA-microRNA-mRNA axis can represent a potential therapeutic alternative in OC treatment to overcome the mechanisms of resistance through combination therapy.

## 8. Conclusions and Perspectives

OC, known as the “silent killer”, spreads broadly before diagnosis without the manifestation of specific signs. OC symptoms typically resemble some gastrointestinal problems; thus, many OCs are diagnosed when already in an advanced stage. Understanding the mechanisms of OC peritoneal metastasis and discovering new biomarkers are imperative to developing effective strategies for early intervention. With the improvement of precision medicine, the management of OC has evolved from a ‘one-size-fits-all’ approach to personalized medicine, in which pharmaceutical therapy and targeted therapy are designed for a single patient. Although many studies reported the involvement of ncRNAs in OC tumorigenesis and suggested their potential therapeutic role in cancer intervention, more accurate and standardized methods should be developed to screen ncRNAs. Further studies might clarify if different ncRNA expressions according to OC clinicopathological characteristics. CircRNAs, initially considered as splicing-associated noise, currently are considered pivotal gene regulators of several processes, including tumorigenesis. CircRNAs remarkable features include the prevalence, specificity, high stability, and conservation that make them potent biomarkers with diagnostic, prognostic, and therapeutic target values for OC.

EVs and CircRNAs can represent the main actors in deciphering the intricate connection between circRNAs, miRNAs, and mRNAs. The findings on circRNAs have made great progress in recent years, and their application in cancer diagnosis and treatment has shown increasingly potential. However, more comprehensive investigations are needed before circRNAs can be used routinely in clinical practice.

**Figure 6:**
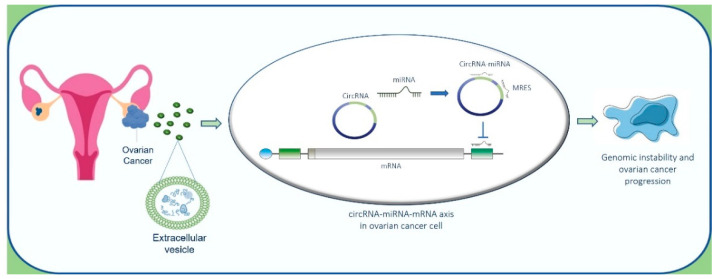
Hypothesis of the role of circRNA-miRNA-mRNA axis in OC progression. Cancer cells release EVs containing circRNAs sponging miRNAs that, in turn, modulate gene expression inducing genetic instability and tumor progression through epigenetic modifications.

Moreover, several reports indicate that epigenetic modifications can induce genomic instability and cancer progression. As also indicated by our in-silico analysis, the alteration of specific circRNA-microRNA-mRNA axis promotes the genomic instability of OC cells (Figure 6). In this scenario, further studies are needed to investigate the intriguing miRNA-circRNA code to translate this knowledge into new therapeutic options and clinical management. Moreover, circRNA–microRNA code can represent an alternative, valid and reliable source of diagnostic biomarkers with therapeutic relevance that deserve to be further validated in the pre-clinical and clinical settings. Although this field may seem to be in infancy, the data reported in this review indicate the growing potential of ncRNAs as biomarkers and therapeutic targets for precision oncological treatments. The combined analysis of different LB elements, including EVs and ncRNAs, may help to comprehend the dynamics of molecular alterations, supporting the clinical decisions in OC management.

## Figures and Tables

**Table 1:** The circRNAs frequently found upregulated in OC.

CircBank ID	Gene Name	References
Circ-WHSC1	WHSC1	Gan et al. [134]
Circ-MUC16	MUC16	Gan et al. [134]
Circ-Foxo3	Foxo3	Wang et al. [135]
Circ-RNA051239	-	Ma et al. [136]
Circ-0001068	MGAT5	Wang et al. [137]
ciRS-7	Cdr1as	Zhao et al. [138]
Circ-HIPK3	HIPK3	Zhou et al. [139]
Circ_0061140	-	Chen et al. [140]
Circ_0051240	CEACAM5	Zang et al. [141]
Circ-CSPP1	CSPP1	Li et al. [142]
Circ-EPSTI1	EPSTI1	Xie et al. [143]
Circ-PIP5K1A	PIP5K1A	Sun et al. [144]
Circ-GFRA1	GFRA1	Liu et al. [145]
Circ-PLEKHM3	PLEKHM3	Zhang et al. [146]
Circ_0078607	-	Zhang et al. [147]
Circ-EXOC6B	EXOC6B	Wang et al. [148]
Circ-ITCH	ITCH	Luo et al. [120]
Circ_0025033	FOXM1	Huang et al. [152]

**Table 2:** miRNAs common between the 1344 miRNAs sponged by CircRNAs and the 69 miRNAs downregulated in OC patients.

miRNAs Downregulated in OC Cells	Identification in Clinical Samples	References
hsa-miR-124-3p	11 pairs of OC tissues and adjacent non-tumor tissue	Zhang et al. [171]
hsa-miR-1271-5p	18 pairs of OC tissues and adjacent non-tumor tissue	Liu et al. [172]
hsa-miR-132-3p	40 OC samples and 40 normal samples	Jiang et al. [173]
hsa-miR-138-5p	47 pairs of OC tissues and adjacent non-tumor tissue	Qu et al. [174]
hsa-miR-139-5p	46 pairs of OC tissues and adjacent non-tumor tissue	Wang et al. [175]
hsa-miR-143-3p	35 pairs of OC tissues and adjacent non-tumor tissue	Zhang et al. [176]
hsa-mir-153-3p	60 pairs of OC tissues and adjacent non-tumor tissue	Zhou et al. [177]
hsa-miR-16-5p	333 OC samples and 39 normal samples	Singh et al. [178]
hsa-miR-193a-3p	31 pairs of OC tissues and adjacent non-tumor tissue	Wang et al. [179]
hsa-mir-216b-5p	152 OC samples and 2 normal samples	Pei et al. [180]
hsa-mir-218-5p	48 pairs of OC tissues and adjacent non-tumor tissue	Pei et al. [180]
hsa-mir-298	100 OC samples and 20 normal samples	Zhou et al. [181]
hsa-mir-31-5p	85 OC samples and 60 normal samples	Kumar et al. [182]
hsa-mir-335-5p	55 OC samples and 17 normal samples	Cao et al. [183]
hsa-mir-340-5p	10 pairs of OC tissues and adjacent non-tumor tissue	Li et al. [184]
hsa-mir-377-3p	44 pairs of OC tissues and adjacent non-tumor tissue	Yu et al. [185]
hsa-mir-488-3p	27 pairs of OC tissues and adjacent non-tumor tissue	Yang et al. [186]
hsa-mir-494-5p	96 pairs of OC tissues and adjacent non-tumor tissue	Yang et al. [187]
hsa-miR-497-5p	126 pairs of OC tissues and adjacent non-tumor tissue	Wang et al. [188]
hsa-mir-508-5p	84 pairs of OC tissues and adjacent non-tumor tissue	Hong et al. [189]
hsa-mir-613	30 pairs of OC tissues and adjacent non-tumor tissue	Fu et al. [190]
hsa-mir-654-5p	107 OC samples and 29 normal samples	Majem et al. [191]
hsa-mir-655-3p	28 OC samples and 15 normal samples	Liu et al. [192]
hsa-mir-708-5p	243 OC samples and 28 normal samples	Lin et al. [193]
